# The association between homocysteine levels and cardiovascular disease risk among middle-aged and elderly adults in Taiwan

**DOI:** 10.1186/s12872-021-02000-x

**Published:** 2021-04-20

**Authors:** Chin-Chuan Shih, Yu-Lin Shih, Jau-Yuan Chen

**Affiliations:** 1 General Administrative Department, United Safety Medical Group, 2F, No.302, Zhongzheng Rd., Xinzhuang District, New Taipei City, 242 Taiwan (R.O.C.); 2 Department of Family Medicine, Chang-Gung Memorial Hospital, Linkou Branch, No.5, Fuxing St., Guishan Dist, Taoyuan City, 333 Taiwan (R.O.C.); 3 Chang Gung University College of Medicine, Taoyuan, No.259, Wenhua 1st Rd., Guishan Dist, Taoyuan City, 333 Taiwan (R.O.C.)

**Keywords:** Homocysteine, Cardiovascular disease, Middle-aged and elderly, Framingham risk score

## Abstract

**Background:**

Our study aimed to determine the association between homocysteine levels and cardiovascular disease (CVD) risk in middle-aged and elderly adults in a community in northern Taiwan.

**Methods:**

Participants in our study included adults aged 50 to 85 years old during community health examinations in 2019. A total of 396 people were enrolled, the ethnicity of all participants is Chinese. We divided participants according to tertiles of ln[homocysteine] level (low, middle and high groups). The CVD risk was calculated by the Framingham cardiovascular risk score (FRS). An FRS ≥ 20% indicated high CVD risk. Pearson correlation coefficients were calculated between homocysteine level and other cardio-metabolic risk factors while adjusting for age. Multivariate logistic regression analysis was used to determine the association of high and middle ln[homocysteine] groups with high CVD risk after adjusting age, sex, uric acid, creatinine, and body mass index (BMI). The Youden index and receiver operating characteristic (ROC) curves were performed to determine the optimized cut-off value.

**Results:**

There were 396 people enrolled for analysis; 41.4% of participants were male, and the average age was 64.79 (± 8.76). In our study, we showed a positive correlation of homocysteine with FRS. In the logistic regression models, higher ln[homocysteine] levels was associated with higher CVD risk with a odds ratio (OR) of 2.499 and 95% confidence interval (CI) of 1.214 to 5.142 in the high homocysteine level group compared with the low homocysteine group after adjusting for traditional CVD risk factors. The area under the ROC curve was 0.667, and a ln[homocysteine] cut-off value of 2.495 µmol/L was determined.

**Conclusions:**

Middle-aged and elderly people with increased homocysteine levels were associated with higher FRSs in this Taiwan community. Furthermore, homocysteine was an independent risk factor for high CVD risk in this study.

## Background

Despite progress in medicine, the National Center for Health Statistics revealed that heart disease is still the leading cause of death in the United States [1]. The World Health Organization has also indicated that cardiovascular diseases (CVDs) are the number one cause of death globally [2], causing great loss and heavy burden in society [3]. Several CVD risk prediction models have been developed, and the Framingham Heart Study has been the most famous system for developing these prediction models [4]. Health care providers are always interested in finding reliable biomarkers for CVD.

Endothelial damage and inflammation initiate the series of steps in CVD development [5], and many biomarkers related to those steps have been investigated. C-reactive protein (CRP) has emerged as a potential useful marker for CVD, and chronic vessel inflammation suggests the possibility that subclinical states can be identified by an increasing CRP level as an indicator of circulating markers of inflammation before acute events occur [6]. The erythrocyte sedimentation rate (ESR) is another potentially novel biomarker to predict CVD, and a study showed that the ESR was mostly prolonged in patients with coronary heart disease [7].

Homocysteine is a nonproteinogenic α-amino acid in the human body and cannot be obtained from the diet. Instead, homocysteine is synthesized from methionine via multiple biochemical steps. Homocysteine can be transformed into L-cysteine or back into L-methionine. Bacteria and plants have different pathways to produce homocysteine.

An elevated homocysteine level was considered to be a risk factor for CVD in recent studies [8]. It has been believed that homocysteine disrupts endothelial function, leading to vessel damage and, ultimately, to CVD [9, 10].

Previous studies focused on mechanisms and relationships, but in our study, we wanted to know whether homocysteine is an independent risk factor for CVD. We collected many parameters related to CVD, including the traditional cardio-metabolic risk factors, in this study. After analysis, our findings revealed that homocysteine was an independent risk factor for high CVD risk.

## Methods

### Study design and participants

**Fig. 1:**
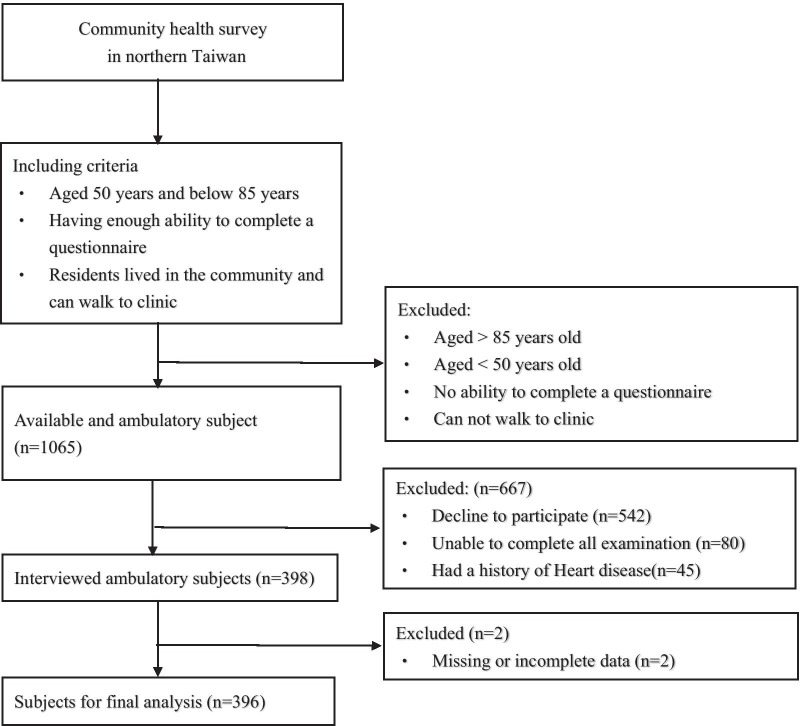
Flow chart of study subjects

This was a cross-sectional and community-based study with participants from a community health survey project conducted in 2019 in northern Taiwan. The inclusion criteria were as follows: (1) aged 50 years and below 85 years; (2) ability to complete a questionnaire; (3) living in the community and ability to walk to the clinic; (4) completion of all examinations. The exclusion criteria were (1) a history of heart disease or (2) missing or incomplete data (Fig. 1). Finally, 396 subjects were enrolled in this study and eligible for analysis. Each participant completed a questionnaire including personal information and medical history during face-to-face interviews. This study was approved by the Institutional Review Board (IRB) of Linkou Chang Gung Memorial Hospital, and all participants were informed and gave their consent before enrollment. All the participants signed consent form before entering the study.

### Data collection and measurements

The content of the questionnaire included sex, age, alcohol drinking status (no drinking, occasional drinking, drinking ≥ 3 days/week, or former drinker) and current smoking (self-reported current smoker, former smoker or nonsmoker). The health survey collected vegetarian diet, hypertension (HTN), diabetes mellitus (DM), and dyslipidemia data. Resting systolic and diastolic blood pressure (BP, mmHg) were measured at least two times at rest. The following biochemical laboratory parameters were analyzed at the Roche® model lab at Taiwan E&Q Clinical Laboratory: fasting plasma glucose (FPG), homocysteine, low-density lipoprotein (LDL-C, mg/dl), high-density lipoprotein (HDL-C, mg/dl), total cholesterol (TC, mg/dl), triglyceride level (TG, mg/dl), alanine transaminase (ALT, mg/dl), creatinine (mg/dl), and uric acid (mg/dl). Waist circumference (WC, cm) was measured at the midpoint between the inferior margin of the last rib and the iliac crest in a horizontal plane while the individual was in an upright position. Body mass index (BMI) was calculated as the person's weight in kilograms divided by the square of height in meters.

### Definition of CVD risk and other variables

In this study, the CVD risk was defined according to the 2008 general CVD risk model from the Framingham Heart Study, which included age, sex, systolic blood pressure (SBP), anti-HTN medication, current smoking, DM, TC, and HDL-C [13]. According to the previous study, the participants can be categorized into low-risk group (Framingham Risk Score [FRS] < 10%), a middle-risk group (10% ≤ FRS < 20%), and a high-risk group (FRS ≥ 20%) [11], so we classified our participants into two categories: high CVD risk (FRS ≥ 20%) and non-high CVD risk (FRS < 20%) for logistic regression. DM was defined as an FPG ≥ 126 mg/dL or the use of oral hypoglycemic agents or insulin therapy. HTN was defined as SBP ≥ 140 mmHg, diastolic blood pressure (DBP) ≥ 90 mmHg, or the use of treatment for HTN. Dyslipidemia was defined as LDL-C ≥ 130 mg/dL, HDL-C < 40 mg/dL in males or < 50 mg/dL in females, TG ≥ 150 mg/dL, TC ≥ 200 mg/dL, or the use of lipid-lowering medication.

### Statistical analysis

In order to normalize our data, we used Ln-transformation for homocysteine level and FRS. Participants were divided into three groups according to ln[homocysteine] level: low ln[homocysteine] level (< 2.42), middle ln[homocysteine] level (2.42–2.66), and high ln[homocysteine] level (≥ 2.67). For laboratory and clinical data within each group, continuous variables were expressed as the mean ± SD and analyzed by one-way Analysis of variance (ANOVA); categorical variables were expressed as n (%) and analyzed by chi-square test. Pearson’s correlation coefficient was used to analyze correlations between ln[homocysteine] and age, ln[FRS], FPG, TG, HDL-C, LDL-C, uric acid, WC, SBP, DBP and BMI; the Pearson’s correlation adjusted by age was also presented. The Pearson’s correlation indicated the potential risk factors for us to discuss in more detail in logistic regression analysis. In addition, we further calculated the prevalence of high CVD risk according to the three levels of homocysteine. Finally, each three groups which were determined by the level of the ln[homocysteine] were divided into two sub-groups: high CVD risk subgroup (FRS ≥ 20%) and non-high CVD risk subgroup (FRS < 20%), then a multiple logistic regression was performed to evaluate the association between homocysteine and high CVD risk, for the purpose of binary logistic regression analysis we had mentioned that two groups of outcome variable would be more appropriate. In our study, a *p *value of < 0.05 was considered statistically significant. Youden index, the sum of sensitivity and specificity minus one, was performed. The maximum result of the Youden index decided the optimized ln[homocysteine] cut-off value to determine high CVD risk. We also presented receiver operating characteristic (ROC) curves. All statistical analyses were performed using SPSS for Windows (IBM Corp. Released 2011. IBM SPSS Statistics, version 25.0. Armonk, NY: IBM Corp.).

## Results

Data from middle-aged and elderly people from communities in northern Taiwan were analyzed in this study. A total of 396 individuals, including 164 men (41.4%) and 232 women (58.6%) with a mean age of 63.72 ± 8.76 years, were enrolled for analysis. Table 1 summarizes the demographic and clinical characteristics of the study subjects. The enrolled patients were categorized into three subgroups according to their ln[homocysteine] level group: low ln[homocysteine] level (< 2.42), middle ln[homocysteine] level (2.42–2.66), and high ln[homocysteine] level (≥ 2.66). There were no statistically significant differences in age, TG concentration, LDL-C concentration, ALT concentration, alcohol drinking, prevalence of hyperlipidemia, or vegetarian diet between the low, middle and high ln[homocysteine] level groups. The participants in the high ln[homocysteine] group were more likely to be male and have a higher FPG level, ln[FRS], uric acid concentration, creatinine concentration, waist circumference, SBP, DBP, BMI, smoking rate, prevalence of HTN, and prevalence of DM than those in the low ln[homocysteine] group. In addition, HDL-C was lower among participants in the high ln[homocysteine] group.

**Table 1:** General characteristics of the study population according to tertiles of ln[homocysteine]

Variable	ln[homocysteine]				
	Total	Low	Middle	High	*p* value for trend
		(< 2.42)	(2.42–2.66)	(≥ 2.67)	
	(n = 396)	(n = 132)	(n = 130)	(n = 134)	
Age (year)	64.79 ± 8.76	64.17 ± 8.5	65.32 ± 9.34	64.87 ± 8.47	0.521
Gender (male), n(%)	164(41.4)	24(18.2)	52(40.0)	88(65.7)	< 0.001
Fasting plasma glucose (mg/dL)	109.79 ± 35.65	102.89 ± 20.65	111.74 ± 41.87	114.68 ± 39.74	0.007
FRS (%)	2.61 ± 0.67	2.35 ± 0.62	2.60 ± 0.69	2.87 ± 0.60	< 0.001
ln[homocysteine(umol/L)]	2.56 ± 0.32	2.23 ± 0.15	2.54 ± 0.07	2.90 ± 0.22	< 0.001
TG (mg/dL)	141.07 ± 110.00	137.95 ± 105.33	141.65 ± 130.97	143.59 ± 91.41	0.677
LDL-C (mg/dl)	109.69 ± 33.99	113.27 ± 32.46	108.09 ± 35.97	107.71 ± 33.46	0.183
HDL-C (mg/dl)	53.56 ± 14.51	56.99 ± 13.87	54.42 ± 14.30	49.34 ± 14.41	< 0.001
Uric Acid (mg/dL)	5.63 ± 1.52	5.13 ± 1.27	5.53 ± 1.35	6.23 ± 1.70	< 0.001
ALT (U/L)	27.16 ± 22.82	26.06 ± 25.70	28.15 ± 21.44	27.28 ± 21.18	0.665
Creatinine (mg/dl)	0.87 ± 0.43	0.73 ± 0.14	0.80 ± 0.19	1.07 ± 0.66	< 0.001
waist circumference (cm)	85.36 ± 10.83	81.01 ± 10.06	86.01 ± 10.55	89.00 ± 10.40	< 0.001
SBP (mmHg)	137.30 ± 17.49	134.28 ± 16.60	137.29 ± 18.17	140.29 ± 17.29	0.005
DBP (mmHg)	85.19 ± 10.98	83.61 ± 10.62	84.04 ± 10.35	87.86 ± 11.49	0.001
BMI (kg/m^2^)	25.59 ± 3.84	24.53 ± 3.38	25.49 ± 3.54	26.74 ± 4.23	< 0.001
Smoking, n(%)	50(12.6)	7(5.3)	14(10.8)	29(21.6)	< 0.001
Alcohol drinking, n(%)	28(7.1)	8(6.1)	9(6.9)	11(8.2)	0.495
HTN, n(%)	201(50.8)	55(41.7)	63(48.5)	83(61.9)	0.001
DM, n(%)	133(33.6)	29(22.0)	49(37.7)	55(41.0)	0.001
Hyperlipidemia, n(%)	153(38.6)	49(37.1)	43(33.1)	61(45.5)	0.158
Vegetarian, n(%)	12(3.0)	4(3.0)	3(2.3)	5(3.7)	0.737

Clinical characteristics are expressed as mean ± SD for continuous variables and n(%) for categorical variables. *p *value were derived from one-way analysis of variance (one-way ANOVA) for continuous variables and chi-square test for categorical variables

FRS, Framingham cardiovascular risk score; TG, triglyceride; LDL-C, low-density lipoprotein cholesterol; HDL-C, high-density lipoprotein cholesterol; ALT, alanine aminotransferase; SBP, systolic blood pressure; DBP, diastolic blood pressure; BMI, body mass index; HTN, hypertension; DM, diabetes mellitus; CVD, cardiovascular disease

**Fig. 2:**
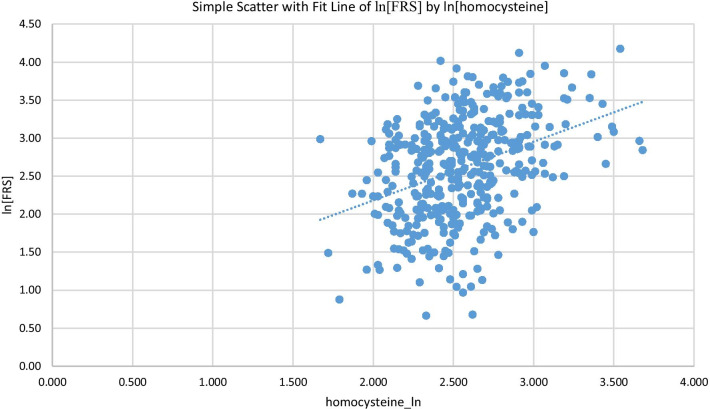
The correlation between ln[homocysteine] and ln[FRS]. FRS, Framingham cardiovascular risk score

Table 2 demonstrates the correlations between ln[homocysteine] level and various cardiovascular risk factors. ln[homocysteine] level was positively correlated with ln[FRS], FPG, uric acid concentration, waist circumference, SBP, DBP, and BMI; ln[homocysteine] level was negatively correlated with HDL-C concentration. Most of these associations remained statistically significant after adjusting for age. Age, TG and LDL-C concentration were not significantly associated with ln[homocysteine]. Figure 2 shows a scatterplot of CVD risk by ln[homocysteine] level. The Pearson’s correlation was 0.368 with a *p *value < 0.001.

**Table 2:** The Pearson correlation between ln[homocysteine] and cardio-metabolic risk factors

Variable	ln[homocysteine]			
	Unadjusted		Adjusted for age	
	Correlation	*p* value	Correlation	*p* value
Age (year)	0.092	0.068	N/A	N/A
ln[FRS (%)]	0.368	< 0.001	0.391	< 0.001
Fasting plasma glucose (mg/dL)	0.103	0.040	0.094	0.063
TG (mg/dL)	0.025	0.618	0.408	0.521
HDL-C (mg/dl)	− 0.216	< 0.001	− 0.495	< 0.001
LDL-C (mg/dl)	− 0.082	0.103	− 0.047	0.348
Uric Acid (mg/dL)	0.351	< 0.001	0.299	< 0.001
waist circumference (cm)	0.287	< 0.001	0.397	< 0.001
SBP (mmHg)	0.152	0.002	0.555	< 0.001
DBP (mmHg)	0.165	0.001	0.484	< 0.001
BMI (kg/m^2^)	0.214	< 0.001	0.325	< 0.001

FRS, Framingham cardiovascular risk score; TG, triglyceride; LDL-C, low-density lipoprotein cholesterol; HDL-C, high-density lipoprotein cholesterol; SBP, systolic blood pressure; DBP, diastolic blood pressure; BMI, body mass index

**Fig. 3:**
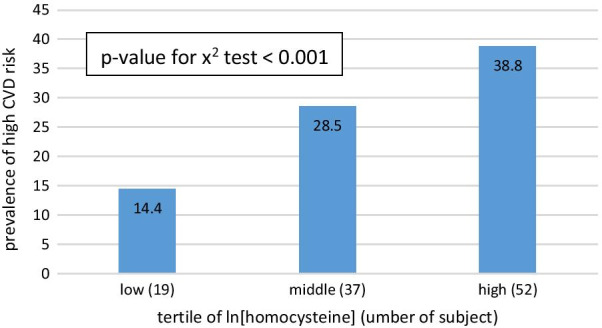
prevalence of high CVD risk based on homocysteine level. A linear increasing trend across homocysteine tertiles. CVD, cardiovascular disease

**Fig. 4:**
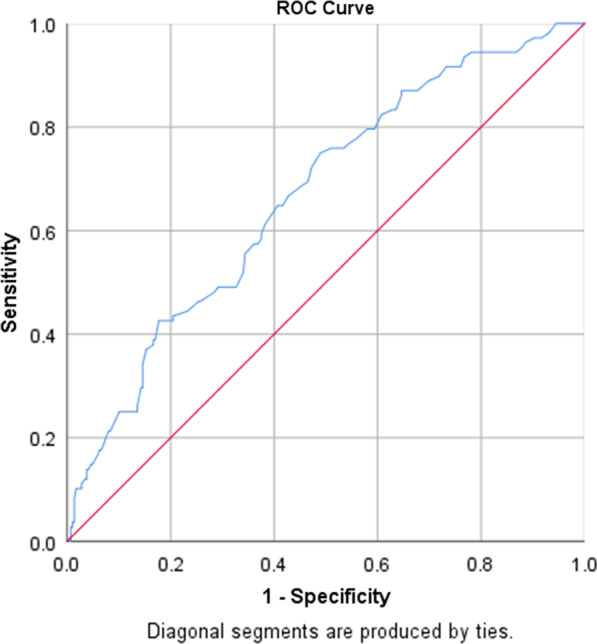
ROC curve for ln[homocysteine] as a biomarker of high CVD risk. CVD, cardiovascular disease

In Table 3, Participants were divided into three groups according to ln[homocysteine] level: low ln[homocysteine] level (< 2.42), middle ln[homocysteine] level (2.42–2.66), and high ln[homocysteine] level (≥ 2.66). Than each groups were further divided into two sub-groups: high CVD risk (FRS ≥ 20) and non-high CVD risk (FRS < 20). Multiple logistic regression models were used to calculate the odds ratio (OR) of ln[homocysteine] levels with high CVD risk after adjustment for other risk factors in each model. The high and middle ln[homocysteine] groups were compared with the low ln[homocysteine] group in all three models. Model 1 was unadjusted; Model 2 was adjusted for age and sex; Model 3 was adjusted for age, sex, uric acid, creatinine and BMI. ln[homocysteine] levels remained statistically significant even after adjusting in Table 3. The OR for high CVD risk was 2.499 (1.214 to 5.142) in the high ln[homocysteine] group compared with the low ln[homocysteine] group in model 3. In Fig. 3, the participants in the high ln[homocysteine] group had higher prevalence of high CVD risk. Figure 4 shows the ROC curve for homocysteine level as a biomarker of high CVD risk. Youden index were used to determine the optimized homocysteine cut-off value to determine high CVD risk. The area under the ROC curve was 0.67; the ln[homocysteine] cut-off value of 2.495 with a sensitivity of 0.75 and a specificity of 0.49 (Table 4), and the Youden index was 0.24.

**Table 3:** association between ln[homocysteine] and high cardiovascular risk

Variable	Model 1			Model 2			Model 3		
	OR	(95% C.I.)	*p* value	OR	(95% C.I.)	*p* value	OR	(95% C.I.)	*p* value
Low	1	–	–	1	–	–	1	–	–
Middle	2.366	1.276 to 4.388	0.006	2.073	1.070 to 4.013	0.031	1.865	0.955 to 3.641	0.068
High	3.772	2.075 to 6.854	< 0.001	3.348	1.709 to 6.559	< 0.001	2.499	1.214 to 5.142	0.013

Model 1: unadjusted

Model 2: unadjusted for model 1 plus age and gender

Model 3: adjusted for model 2 plus uric acid, creatinine, and BMI

BMI, body mass index

**Table 4:** The areas under ROC curve (AUC), sensitivity, specificity by the optimized cut-off points for ln[homocysteine] as a biomarker of high cardiovascular risk

Variable	Area	*p* value	Cut-off point	Sensitivity	Specificity
ln[homocysteine]	0.667(0.608–0.726)	< 0.000	2.495	0.75	0.49

## Discussion

CVD has been a worldwide healthcare burden for a long time, and medical providers are always interested in finding reliable biomarkers for CVD and CVD risk factors. In this community-based study, we investigated homocysteine levels in association with high CVD risk in middle-aged and elderly people in northern Taiwan. Looking at three ln[homocysteine] tertiles (Table 1), there was a rising proportion of males, HTN, smoking status, and SBP as the ln[homocysteine] level increased. These results correspond with previous studies, [12–14] but HDL had an inverse relationship with ln[homocysteine], which was also noted in a previous study [15]. According to the Framingham risk score, male sex, HTN, smoking status, and SBP are risk factors for CVD, but HDL serve as a protective factor against CVD. [16] Meanwhile, an increase in ln[homocysteine] level is directly associated with increases in ln[FRS]. In Fig. 3, the prevalence of high CVD risk was significantly greater in the higher tertiles of ln[homocysteine]. These findings led us to speculate that an association exists between homocysteine levels and FRS.

In Table 2, we found a positive correlation between ln[homocysteine] levels and ln[FRS], along with other traditional CVD risk factors such as BMI and waist circumference. Most the correlations reached statistical significance even after adjustment for age. The Pearson’s correlation between ln[homocysteine] and ln[FRS] was 0.391 with a p value < 0.001, which corresponds with a previous study that homocysteine can be a risk factor for CVD [7, 17]. All the previous results raise the question of whether homocysteine can be an important risk factor for predicting high CVD risk. Hence, we wanted to know whether ln[homocysteine] can be an independent risk factor for high CVD risk or not. In the next step, we used logistic regression model to see how the homocysteine affects CVD risk.

In the logistic regression models (Table 3), the middle and high ln[homocysteine] groups were compared with the low ln[homocysteine] group, and the prevalence of high CVD risk increased as ln[homocysteine] increased. After adjusting for age, sex, uric acid, creatinine and BMI, the odds ratio (OR) and 95% confidence interval for high CVD risk was 2.499 (1.214 to 5.142) in the high ln[homocysteine] group compared with the low ln[homocysteine] group. This result confirmed that ln[homocysteine] was an independent risk factor for high CVD risk.

Figure 4 shows the ROC curve for ln[homocysteine] as a biomarker for high CVD risk. The AUC was 0.667. In Table 4, Youden index were used to determine the optimized homocysteine cut-off value to determine high CVD risk. The Youden index was 0.24 and the optimized cut-off value for ln[homocysteine] was 2.495 with a sensitivity of 0.75 and a specificity of 0.49. Although this result was not sensitive enough for screening test and was not specific enough for diagnostic test, the result still obviously indicated the positive relationship between ln[homocysteine] and high CVD risk.

There are many possible explanations for the relationship between homocysteine and CVD. Homocysteine is a sulfur-containing amino acid that is a metabolic precursor of methionine and cystathionine [18]. Elevated homocysteine levels are not only considered a risk factor for cardiovascular disease [8, 16, 18] but are also linked to various diseases, such as chronic kidney disease [19]. A previous animal study with cell culture showed that high homocysteine levels had toxic effects on the vasculature, with manifestations of medial remodeling, adventitial inflammation, and endothelial injury. The mechanisms include abnormal protein metabolism [20] and the production of reactive oxidative species [21, 22]. Homocysteine specifically damages the endothelial, medial and adventitial layers of the vessel wall [9], than vessel damage leads to atherosclerosis [23], hypertension [24], stroke [25], coronary artery disease [26], peripheral arterial disease [27], and aneurysm [28].

Many studies have pointed out the positive relationship between homocysteine and CVD, but most of them did not consider the influence of other traditional CVD risk factors on the relationship between homocysteine and CVD. In our study, we considered traditional CVD risk factors and other parameters, and the results revealed that homocysteine level may be an important independent predictor of high CVD risk. Our findings may have an impact on health screening among middle-aged and elderly populations. In addition to those traditional parameters of CVD, homocysteine level should be considered an important biomarker in health screening, and people with elevated homocysteine should be warned of higher CVD risk.

However, our study still had limitations. We recruited participants from northern Taiwan as our favored population. The characteristics of those participants might differ from characteristics in the general population, the selection bias should be considered, so the findings cannot be generalized to the whole middle-aged and elderly population in Taiwan. The results of our study should not be extrapolated to other regions of Taiwan. Future studies using random sampling of communities with a wider range of regions would make the research more discursive.

## Conclusions

This study showed that increased homocysteine levels are associated with high CVD risk among middle-aged and elderly populations in Taiwan. After this cross-sectional study examining the relationship between homocysteine and CVD risk, further follow-up and cohort studies are required to determine the effect of homocysteine on CVD.

## Data Availability

The datasets used and/or analysed during the current study are available from the corresponding author on reasonable request.

## Abbreviations

CVD: Cardiovascular disease
FRS: Framingham cardiovascular risk score
HTN: Hypertension
DM: Diabetes mellitus
BMI: Body mass index
ROC: Receiver operating characteristic
OR: Odds ratio
CVDs: Cardiovascular diseases
CRP: C-reactive protein
ESR: Erythrocyte sedimentation rate
IRB: Institutional Review Board
BP: Blood pressure
FPG: Fasting plasma glucose
LDL-C: Low-density lipoprotein
HDL-C: High-density lipoprotein
TC: Total cholesterol
TG: Triglyceride level
ALT: Alanine transaminase
WC: Waist circumference
BMI: Body mass index
DBP: Diastolic blood pressure
SBP: Systolic blood pressure
ANOVA: Analysis of variance
AUC: Areas under ROC curve
